# Aplastic anemia following the severe acute respiratory syndrome coronavirus‐2 vaccine

**DOI:** 10.1002/jha2.602

**Published:** 2022-11-04

**Authors:** Rodrick Babakhanlou, Tapan Kadia, Kelly Chien, Koji Sasaki, Philip A. Thompson

**Affiliations:** ^1^ Department of Leukemia, MD Anderson Cancer Center The University of Texas Houston Texas United States

1

To the Editor,

There have been several reports describing the possible relationship between the severe acute respiratory syndrome coronavirus‐2 (SARS‐CoV‐2) vaccine and the development of hematological diseases, such as autoimmune hemolytic anemias, paroxysmal nocturnal hemoglobinuria or immune thrombocytopenic purpura [1, 2, 3, 4, 5, 6]. Although a few reports have mentioned a potential relationship between the SARS‐CoV‐2 vaccine and the development of aplastic anemia (AA), data is still limited [7, 8, 9, 10].

Herein, we report four patients seen at our institution, who were diagnosed with AA after receiving the SARS‐CoV‐2 vaccine (Table 1).

**TABLE 1:** Characteristics of four patients with aplastic anemia following the severe acute respiratory syndrome coronavirus‐2 (SARS‐CoV‐2) vaccine at initial presentation

**Patient**	**1**	**2**	**3**	**4**
Age	68	60	19	50
Gender	Male	Male	Male	Male
Hemoglobin (g/dl) (14–18 g/dl)	5.8	9.2	7.9	4.3
MCV (fl) (82–98 fl)	99	98	95	96
Platelets (x10^9^/L) (140–440 K/μl)	8	4	17	0
WBC (x10^9^/L) (4–11 K/μl)	3	4.5	0.6	3.5
Absolute neutrophils (K/μl) (1.7–7.3 K/μl)	0.75	3.83	0.01	0.39
Absolute reticulocyte count (x10^9^/L)	28.6	44.64	1.1	10
Bone marrow cellularity (%)	5–10	10	5	10–20
Severity category of aplastic anemia [8]	Severe	Severe	Very severe	Severe
Direct anti‐globulin test (DAT)	Negative	Negative	Negative	Negative

**Patient 1**: A 68‐year‐old man with no significant past medical history received the Moderna mRNA‐1273 vaccine with the first dose in January 2021 and the second dose in February 2021. Four weeks after receiving the second dose of the Moderna mRNA‐1273 vaccine he developed dizziness, fatigue, chest tightness, dyspnea, and nose bleeds. His laboratory results on presentation to our institution are outlined in Table 1. The bone marrow was acellular. His cytogenetics showed a diploid karyotype and there were no chromosomal abnormalities. There were no mutations on an 81‐gene myeloid‐focused next‐generation sequencing panel. He was diagnosed with AA and treated with combination immunosuppressive therapy consisting of methylprednisolone, horse anti‐thymocyte globulin (ATG), cyclosporine, and eltrombopag, achieving partial remission. He has been transfusion‐independent since February 2022, but still receives treatment with eltrombopag 150 mg daily, cyclosporine 100 mg daily, and Danazol 400 mg daily, which was introduced in November 2021 due to side effects of eltrombopag. His best laboratory values achieved were 14 months after initiation of therapy and included a Hb of 11.3 g/dl with a mean corpuscular volume (MCV) of 110 fl, a platelet count of 61 × 10^9^/L, and an ANC of 1.15 K/μl.

**Patient 2**: A 60‐year‐old man with no significant past medical history received the Pfizer‐BioNTech vaccine with the first dose in March 2021 and the second dose in April 2021. Two weeks after the second dose he developed fatigue, bruising and petechial bleeding. His laboratory results on presentation to our institution are outlined in Table 1. Haptoglobin was 112 mg/dl. His bone marrow had cellularity of 10%. Cytogenetics showed a diploid karyotype and there were no chromosomal abnormalities. There were no mutations on an 81‐gene myeloid‐focused next‐generation sequencing panel. He was diagnosed with AA and received treatment with a combination immunosuppressive therapy consisting of methylprednisolone, horse ATG, cyclosporine, and eltrombopag. His blood counts started recovering after approximately three months of therapy and he has remained transfusion‐independent and in complete remission since then. His best laboratory values achieved were 6 months after initiation of therapy and included a Hb of 14.3 g/dl with an MCV of 98 fl, a platelet count of 159 × 10^9^/L and an ANC of 1.9 K/μl. He currently takes cyclosporine 100 mg twice daily.

**Patient 3**: A 19‐year‐old man with a past medical background of type 1 diabetes received the Pfizer‐BioNTech vaccine. The first dose was given in April 2021, followed by the second dose in May 2021. Two weeks after the second dose he developed headaches, fever, fatigue, petechial bleeding, and gingival bleeding. His laboratory results on presentation to our institution are outlined in Table 1. The bone marrow was acellular. His cytogenetics showed a diploid karyotype and there were no chromosomal abnormalities. There were no mutations on an 81‐gene myeloid‐focused next‐generation sequencing panel. He was diagnosed with AA and received treatment with a combination immunosuppressive therapy consisting of methylprednisolone, horse ATG, eltrombopag, and granulocyte colony‐stimulating factor (G‐CSF). His symptoms improved two months after the initiation of treatment, and he recovered after three months achieving complete remission. His best laboratory results achieved three months after initiation of therapy included a Hb of 12.8 g/dl with an MCV of 96 fl, a platelet count of 141 × 10^9^/L and an ANC of 2.00 K/μl. He currently takes cyclosporine 200 mg twice daily.

**Patient 4**: A 50‐year‐old male patient received the Moderna mRNA‐1273 vaccine with the first dose given in February 2021, followed by the second dose in March 2021. Four weeks after having received the second dose, the patient developed bruising and shortness of breath. His laboratory results on presentation to our institution are outlined in Table 1. His cytogenetics showed a diploid karyotype and there were no chromosomal abnormalities. There were no mutations on an 81‐gene myeloid‐focused next‐generation sequencing panel. He was diagnosed with AA and received treatment with a combination immunosuppressive therapy consisting of methylprednisolone, horse ATG, cyclosporine, eltrombopag, and G‐CSF. His symptoms started improving in January 2022 and he has remained transfusion‐independent since February. He has achieved complete remission and his best laboratory values achieved were 16 months after initiation of therapy and included a Hb of 14.5 g/dl with an MCV of 96 fl, a platelet count of 266 × 10^9^/L and an ANC of 3.4 K/μl. He is currently on cyclosporine 100 mg daily. This patient contracted coronavirus disease (COVID) in December 2020 with minor symptoms only and had a complete recovery from it within 1 week. Upon arrival at the MD Anderson Cancer Center, his COVID test was negative.

In our reported cases all patients were male with a median age of 56 years (range, 19–69). Two patients (50%) had received the Pfizer‐BioNTech vaccine and two (50%) the Moderna mRNA‐1273 vaccine. One of the patients had contracted COVID 8 weeks prior to his vaccination. One patient suffered from type 1 diabetes. None of the four patients developed any symptoms after the first dose of the vaccine. Symptoms occurred on average 3 weeks after the second vaccine, which is similar to other potentially immune‐related phenomena [6]. All patients had a diploid karyotype. Flow cytometry and molecular diagnostics did not show any abnormalities. In this way, these patients appeared typical of patients with immunologically mediated AA.

No patient had evidence of myelodysplastic syndrome, paroxysmal nocturnal hemoglobinuria, congenital bone marrow failure syndrome, or hemolytic anemia.

None of those patients was known to have had any exposure to occupational substances or medical agents known to cause AA. All patients received combination immunosuppressive therapy with methylprednisolone, horse ATG, cyclosporine, and eltrombopag, three on a clinical trial and one‐off protocol. Patients were on therapy for a median of 26 weeks (16–48). All patients responded to treatment: three patients achieved a complete remission at 12 weeks, while one patient is in partial remission. All patients are transfusion‐independent at the time of this report. This pattern of response appears similar to that seen in patients with idiopathic, presumed immune‐mediated AA. No patient required allogeneic stem cell transplantation.

Although we cannot prove a causative relationship, in rare circumstances, the SARS‐CoV‐2 vaccine could be associated with the development of AA.

## CONFLICT OF INTEREST

The authors declare they have no conflicts of interest.

## FUNDING INFORMATION

The authors received no specific funding for this work.

## Data Availability

The data that support the findings of this study are available in the attached Table 1.
